# Minor prion substrains overcome transmission barriers

**DOI:** 10.1128/mbio.02721-24

**Published:** 2024-10-23

**Authors:** Benjamin S. Steadman, Jifeng Bian, Ronald A. Shikiya, Jason C. Bartz

**Affiliations:** 1 Department of Medical Microbiology and Immunology, School of Medicine, Creighton University, Omaha, Nebraska, USA; 2 Virus and Prion Research Unit, National Animal Disease Center, United States Department of Agriculture – Agricultural Research Services, Ames, Iowa, USA; National Institutes of Health, Bethesda, Maryland, USA

**Keywords:** prion disease, prion strain, interspecies transmission

## Abstract

Mammalian prion diseases are infectious neurodegenerative diseases caused by the self-templating form of the prion protein PrP^Sc^. Much evidence supports the hypothesis that prions exist as a mixture of a dominant strain and minor prion strains. While it is known that prions can infect new species, the relative contribution of the dominant prion strain and minor strains in crossing the species barrier is unknown. We previously identified minor prion strains from a biologically cloned drowsy (DY) strain of hamster-adapted transmissible mink encephalopathy (TME). Here we show that these minor prion strains have increased infection efficiency to rabbit kidney epithelial cells that express hamster PrP^C^ compared to the dominant strain DY TME. Using protein misfolding cyclic amplification (PMCA), we found that the dominant strain DY TME failed to convert mouse PrP^C^ to PrP^Sc^, even after several serial passages. In contrast, the minor prion strains isolated from biologically cloned DY TME robustly converted mouse PrP^C^ to PrP^Sc^ in the first round of PMCA. This observation indicates that minor prion strains from the mutant spectra contribute to crossing the species barrier. Additionally, we found that the PMCA conversion efficiency for the minor prion strains tested was significantly different from each other and from the short-incubation period prion strain HY TME. This suggests that minor strain diversity may be greater than previously anticipated. These observations further expand our understanding of the mechanisms underlying the species barrier effect and has implications for assessing the zoonotic potential of prions.

**IMPORTANCE:**

Prions from cattle with bovine spongiform encephalopathy have transmitted to humans, whereas scrapie from sheep and goats likely has not, suggesting that some prions can cross species barriers more easily than others. Prions are composed of a dominant strain and minor strains, and the contribution of each population to adapt to new replicative environments is unknown. Recently, minor prion strains were isolated from the biologically cloned prion strain DY TME, and these minor prion strains differed in properties from the dominant prion strain, DY TME. Here we found that these minor prion strains also differed in conversion efficiency and host range compared to the dominant strain DY TME. These novel findings provide evidence that minor prion strains contribute to interspecies transmission, underscoring the significance of minor strain components in important biological processes.

## INTRODUCTION

Prions are infectious proteins that result in inevitably fatal neurodegenerative diseases (1). Human prion diseases include kuru, Gerstmann-Straussler-Scheinker syndrome, fatal familial insomnia, variably protease-sensitive prionopathy, and sporadic, genetic, and iatrogenic forms of Creutzfeldt-Jakob disease (CJD) (2, 3). Prion diseases in other mammals range from scrapie in sheep and goats to bovine spongiform encephalopathy (BSE) in cattle and chronic wasting disease (CWD) in cervids (4). Importantly, interspecies transmission of atypical and classical forms of BSE resulted in the following additional prion diseases: transmissible mink encephalopathy (TME), exotic ungulate encephalopathy, feline spongiform encephalopathy, prion disease in non-human primates, and significantly, zoonotic transmission to humans, referred to as variant CJD (5–18). To date, interspecies transmission of scrapie to humans has not been documented, and the zoonotic potential for emerging CWD strains in both North America and Nordic countries is poorly understood (19–24).

Prion strains are operationally defined as strain-specific self-templating PrP^Sc^ conformations which cause heritable disease phenotypes that are encoded by the conformation of PrP^Sc^ (25, 26). Prion strains can differ in titer, incubation periods, clinical signs, distribution of spongiform degeneration in the central nervous system, and host range (27, 28, 29). Strain-specific PrP^Sc^ properties include conformational stability, proteinase resistance, migration on SDS-PAGE, glycosylation patterns, and PrP^Sc^ distribution and deposition patterns between and within tissues (30–34). Recently, PrP^Sc^ structural differences between prion strains have been directly observed via cryo-electron microscopy (35–38). The relationship between strain-specific conformations of PrP^Sc^ and disease phenotype is unknown.

Prions exist as strain mixtures. In natural cases of CJD and scrapie, more than one prion strain has been observed within a single host based upon identification of mixtures of strain-specific PrP^Sc^ properties (39–45). In experimental settings, mixtures of prion strains can lead to strain interference, where the dominant strain suppresses prion replication but does not eliminate other minor prion strains (46–53). These experiments led to the hypothesis that removing the suppressive effects of the dominant strain(s) will allow for the emergence of minor prion strains. Consistent with this hypothesis is the observation that drug-resistant prion strains can emerge from anti-prion drug treatment (54–58). Interestingly, removal of the anti-prion drug results in rapid reversion of the prion strain back to a drug-sensitive phenotype (59, 60). This led the authors to hypothesize that prions exist as quasispecies mixtures of similar but not identical conformations of PrP^Sc^ (61, 62). Biochemical and enzymatic treatment of static prion populations resulted in the reduction of the dominant prion strain PrP^Sc^ conformation, allowing for subsequent amplification of the remaining minor strains. This is interpreted as evidence for minor strains pre-existing in the population (63). Overall, prions exist as dynamic mixtures of a dominant strain and minor prion strains.

The mechanism underlying the species barrier effect is incompletely understood. Transmission of prions to a host with a different PrP amino acid sequence can result in an extension of the incubation period compared to transmission to a host with the same sequence (64–66). Subsequent passages in the new PrP sequence can lead to adaptation, a shortening and stabilization of the incubation period, and strain properties (67–69). The exception to this is during non-adaptive prion amplification, where transmission to a new host does not result in adaptation but rather retains tropism for the original host species (70). A single amino acid between the inoculum PrP^Sc^ and host PrP^C^ can have a profound effect on the transmission barrier effect (71–74). It is hypothesized that these differences reduce the efficiency of conversion either by reducing the strength of the interaction between PrP^Sc^ and PrP^C^ and/or by reducing the capacity of PrP^Sc^ to direct the global conformational rearrangement from an alpha helical structure in PrP^C^ to that of a parallel in register intermolecular beta sheet structure of PrP^Sc^ (75–77). The relative contribution of the dominant strain and minor prion strains in overcoming transmission barriers is, however, unknown. Recent work has developed novel methodologies to isolate pre-existing minor prion strains (63). Here, we further investigated intraspecies and interspecies transmission efficiency of dominant and minor prion strains.

## RESULTS

### Minor prion strains isolated from biologically cloned DY TME

A portion of the minor prion strains tested here were previously isolated from biologically cloned drowsy (DY) TME using either the conformational strain selection assay (CSSA) or the proteinase strain selection assay (PSSA) (63). In Gunnels et al., the 4M CSSA material that was serially passaged in hamsters three times is designated in this paper as CSSA1, CSSA2, and CSSA3, respectively, and the PSSA material amplified using protein misfolding cyclic amplification (PMCA) is designated as PSSA-PMCA (Fig. S1, panel A).

Intracerebral (i.c.) inoculation of the PSSA products in hamsters resulted in all (*n* = 5) of the animals developing clinical signs of hyperexcitability and ataxia at 130 ± 3 days post infection (dpi). Brain material from these animals is designated as PSSA1 (Fig. S1, panel A). As a negative bioassay control, inoculation of hamsters (*n* = 5) with PSSA products from uninfected hamsters failed to result in the development of clinical signs of disease at 400 dpi. As a positive bioassay control, hamsters (*n* = 5) were i.c. inoculated with a 10^−4^ dilution of HY TME-infected brain homogenate, resulting in all animals developing clinical signs of hyperexcitability and ataxia at 79 ± 3 dpi. The dominant and minor strains listed above were next examined for PMCA conversion efficiency within and between species.

### Minor prion strains have a higher intraspecies PMCA conversion efficiency compared to the dominant prion strain DY TME

**Fig 1:**
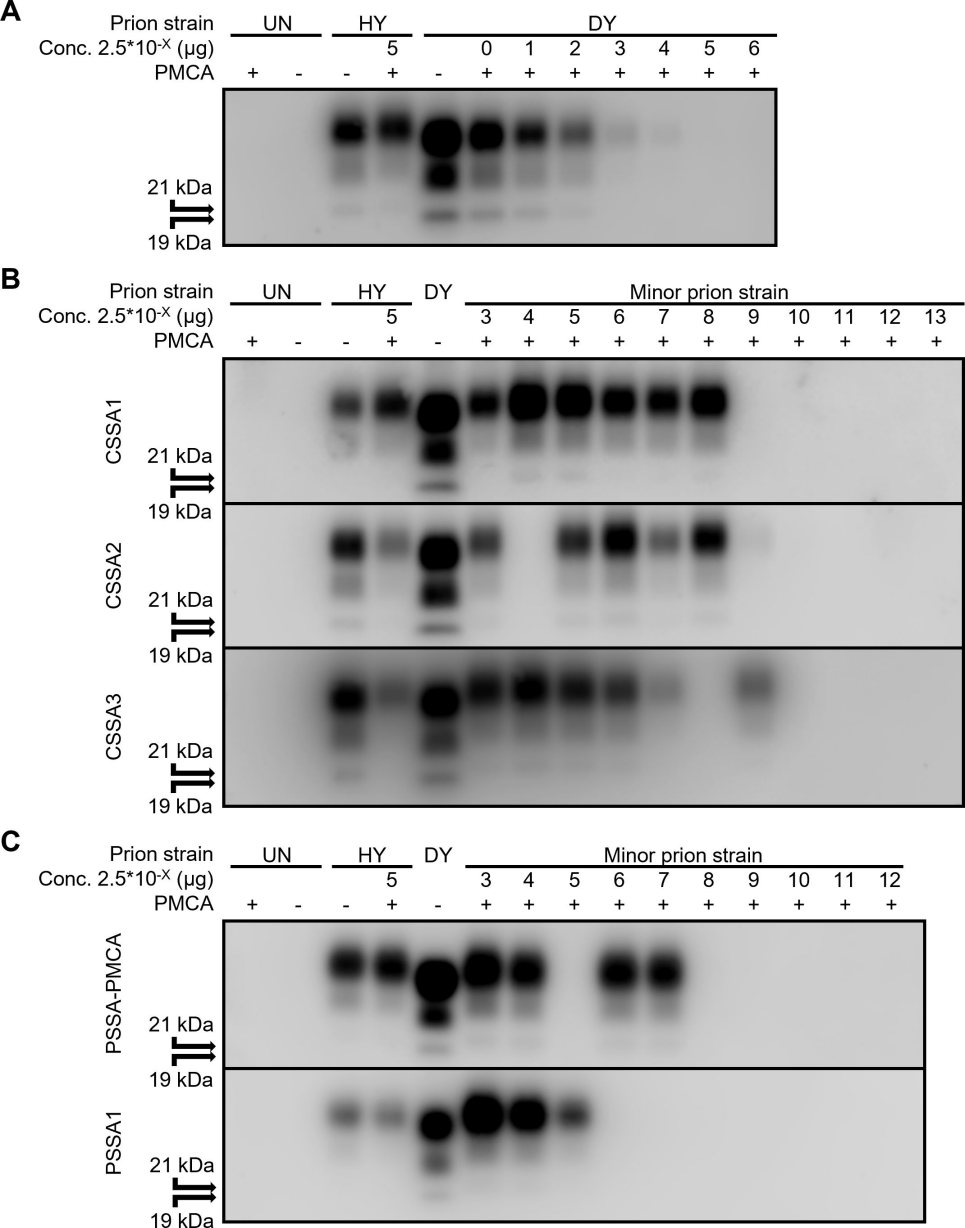
High PMCA conversion efficiency minor prion strains emerge from DY TME. Western blot analysis of intraspecies PMCA reactions seeded with 10-fold serial dilutions of (A) biologically cloned DY TME brain or minor prion strains isolated from DY by either the (B) CSSA or the (C) PSSA. Each prion sample was examined with a minimum of three biological replicates consisting of four technical replicates each. Uninfected (UN) brain homogenate or HY TME-infected brain homogenate seeded PMCA reactions were included in each experiment as negative and positive controls, respectively. CSSA, conformational stability selection assay; PSSA, proteinase strain selection assay.

**Fig 2:**
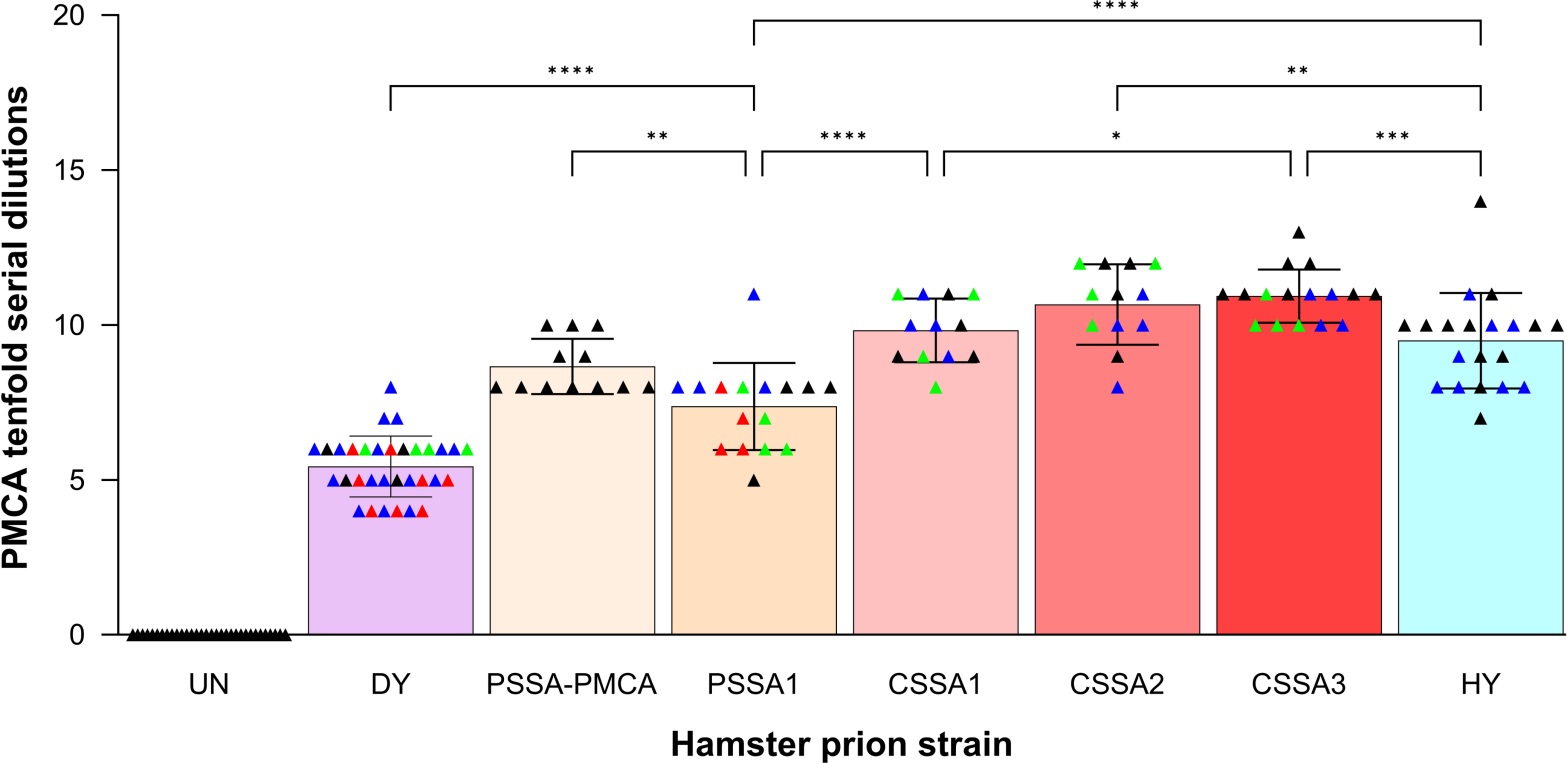
Hamster prion strains differ in PMCA conversion efficiency. Quantification of PMCA conversion efficiency of 10-fold serial dilutions of brain homogenate seeded into intraspecies PMCA reactions. Bar graphs represent all technical replicates for each prion strain, with means and standard deviations. Individual animals for each strain are represented by different colored triangles. *P < 0.05, **P < 0.01, ***P < 0.001, ****P < 0.0001. CSSA1, DY 4M CSSA hamster passage 1; CSSA2, DY 4M CSSA hamster passage 2; CSSA3, DY 4M CSSA hamster passage 3; DY, drowsy hamster-adapted TME; HY, hyper-hamster-adapted TME; PSSA1, DY PSSA hamster passage 1; PSSA-PMCA, DY PSSA hamster PMCA round 4; UN, uninfected hamster.

Intraspecies positive control PMCA reactions were seeded with HY TME-infected hamster brain homogenate into uninfected hamster brain homogenate as the template of conversion. These reactions resulted in Western blot detection of PMCA-generated PrP^Sc^ (Fig. 1). Intraspecies negative control PMCA reactions contained only uninfected hamster brain homogenate. These reactions did not contain detectable PrP^Sc^ by Western blot (Fig. 1). Intraspecies PMCA of 10-fold serial dilutions of brain homogenate from either the dominant strain DY TME (Fig. 1, panel A), the minor strains isolated from DY TME using CSSA (Fig. 1, panel B), or the minor strains isolated from DY TME using PSSA (Fig. 1, panel C) revealed differences in the number of dilutions required until extinction of PrP^Sc^ formation (Fig. 2; Table 1).

**TABLE 1:** Hamster prion strains differ in intraspecies conversion efficiency^*e*^

Hamster prion strain	PMCA conversion efficiency calculation^*a*^		Total PMCA replicate numbers^*d*^
	10-fold serial dilution extinction^*b*^	Detection limit (µg)^*c*^	
Uninfected hamster	0	n.a.	*n* = 147
DY TME	5.4 ± 1.0	9.1 × 10^−4^	*n* = 32
PSSA-PMCA	8.7 ± 0.9	4.4 × 10^−7^	*n* = 12
PSSA1	7.4 ± 1.4	1.1 × 10^−5^	*n* = 16
CSSA1	9.8 ± 1.0	3.7 × 10^−8^	*n* = 12
CSSA2	10.7 ± 1.3	5.4 × 10^−9^	*n* = 12
CSSA3	10.9 ± 0.9	2.9 × 10^−9^	*n* = 16
HY TME	9.5 ± 1.5	7.9 × 10^−8^	*n* = 20
HaCWD	11.3 ± 1.6	1.4 × 10^−9^	*n* = 12
263K scrapie	10.1 ± 1.9	2.1 × 10^−8^	*n* = 12
139H scrapie	6.2 ± 1.6	1.6 × 10^−4^	*n* = 16
ME7H scrapie	3.3 ± 0.7	1.2 × 10^−1^	*n* = 12

^*a*^ The data in Fig. 1 and Fig. S2 were used to determine PMCA conversion efficiency using two different calculations.

^*b*^ The average number of 10-fold serial dilutions of hamster prion strain-infected brain homogenate that resulted in an extinction of prion conversion after one round of PMCA.

^*c*^ The lowest amount of microgram equivalents of hamster prion strain-infected brain homogenate that resulted in detectable amplified PrP^Sc^ following one round of PMCA.

^*d*^ The uninfected samples represent individual technical replicates. All prion strain PMCA replicate numbers represent individual 10-fold serial dilutions of hamster brain homogenate.

^*e*^ n.a., not applicable.

PMCA conversion efficiencies were calculated based on the last dilution of brain homogenate to result in amplification of PrP^Sc^ (Fig. 1) and compared between the dominant strain DY TME and the minor strains (Fig. 2). The PMCA conversion efficiency for DY TME was significantly lower (*P* < 0.0001) compared to all minor strains tested (Fig. 2; Table 1). All CSSA minor strains tested were 4–5 logs more efficient in PMCA conversion efficiency compared to DY TME, and all PSSA minor strains tested were 2–3 logs more efficient in PMCA conversion compared to DY TME (Fig. 2; Table 1). Overall, the minor prion strains isolated from biologically cloned DY TME using either CSSA or PSSA had a greater intraspecies PMCA conversion efficiency than DY TME.

### The PMCA conversion efficiency of minor prion strains differ from each other and from other hamster-adapted prion strains

The PMCA conversion efficiencies were compared between the minor strains isolated from DY TME and other well-characterized hamster-adapted prion strains. Ten-fold serial dilutions of brain homogenate from HY TME-, HaCWD-, 263K scrapie-, 139H scrapie-, and ME7H scrapie-infected brain homogenate were seeded intraspecies into PMCA (Fig. S2) to determine PMCA conversion efficiency (Fig. S3, panel A). The intraspecies PMCA conversion efficiencies were then compared between these known prion strains and the minor strains isolated from DY TME (Fig. 2; Fig. S3, panel A; Table 1). Significant differences were identified between the minor strains isolated from DY TME and other known hamster-adapted prion strains (Fig. 2; Fig. S3, panel A; Table 1). Of note, CSSA2, CSSA3, and PSSA1 intraspecies PMCA conversion efficiency differed significantly (*P* = 0.0076, *P* = 0.0004, *P* < 0.0001, respectively) from HY TME (Fig. 2; Table 1). Overall, the minor prion strains isolated from DY TME using CSSA and PSSA differed in intraspecies PMCA conversion efficiency from each other and from other hamster-adapted prion strains.

### Interspecies PMCA conversion is facilitated by minor prion strains and not the dominant strain

**Fig 3:**
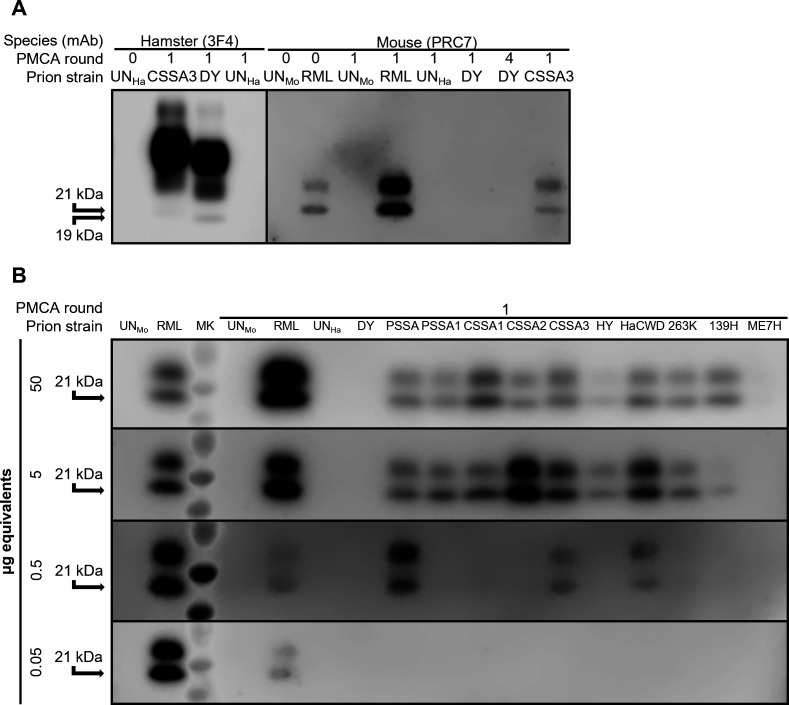
Interspecies prion conversion by minor prion strains. (**A**) Western blot analysis of PMCA reactions using either hamster or mouse as the template for conversion seeded with either UN_Ha_ brain, UN_Mo_ brain, DY TME-infected hamster brain homogenate, the minor prion strain from DY 4M CSSA3, or RML mouse-adapted scrapie. (**B**) Western blot analysis of PMCA reactions seeded with 10-fold serial dilutions (50–0.05 μg eq.) of brain homogenate from uninfected and prion-infected brain homogenates. Each sample was tested by PMCA between three to eight biological replicates consisting of four technical replicates each. CSSA1, DY 4M CSSA hamster passage 1; CSSA2, DY 4M CSSA hamster passage 2; CSSA3, DY 4M CSSA hamster passage 3; PSSA, DY PSSA hamster PMCA round 4; PSSA1, DY PSSA hamster passage 1; RML, Rocky Mountain Laboratory mouse-adapted scrapie; UN_Ha_, uninfected hamster; UN_Mo_, uninfected mouse.

Interspecies PMCA conversion efficiency was compared between the dominant hamster strain DY TME and minor prion strains isolated from DY TME (Fig. S1, panel A) by seeding prion-infected hamster brain into uninfected mouse brain homogenate. As a mouse PMCA positive control reaction, RML-infected mouse brain homogenate was seeded into uninfected mouse brain homogenate, resulting in amplification of PrP^Sc^ (Fig. 3, panel A). Negative control mouse PMCA reactions containing only mouse uninfected brain homogenate or seeded with uninfected hamster brain homogenate failed to amplify PrP^Sc^ (Fig. 3, panel A). Seeding of uninfected mouse brain homogenate with DY TME-infected hamster brain failed to result in PMCA-generated PrP^Sc^ after one or four serial rounds of PMCA (Fig. 3, panel A). This failure of DY TME to convert murine PrP^C^ to PrP^Sc^ was observed from three different DY TME-infected hamster brains that was replicated numerous (*n* = 32) times (Table 2). In contrast, seeding of CSSA3-infected hamster brain homogenate into mouse brain homogenate resulted in detection of PMCA-generated PrP^Sc^ after one round of PMCA (Fig. 3, panel A). As the anti-PrP antibody PRC7 detects mouse PrP, but not hamster PrP (78), we conclude that this is PMCA-generated mouse PrP^Sc^ and not detection of hamster PrP^Sc^ from CSSA3 brain homogenate (Fig. S4).

**TABLE 2:** Prion strains differ in interspecies conversion efficiency after one round of PMCA in mouse brain homogenate^*e*^

Prion strain	PMCA conversion efficiency calculation^*a*^		Total PMCA replicate numbers^*b*^
	10-fold serial dilution extinction^*c*^	Detection limit (µg)^*d*^	
Uninfected mouse	0	n.a.	*n* = 64
RML mouse scrapie	4.5 ± 0.9	0.016	*n* = 20
Uninfected hamster	0	n.a.	*n* = 64
DY TME	0	n.a.	*n* = 32
PSSA-PMCA	2.7 ± 0.8	1.0	*n* = 16
PSSA1	2.0 ± 0.4	5.0	*n* = 16
CSSA1	2.3 ± 1.2	2.8	*n* = 12
CSSA2	2.7 ± 0.6	0.97	*n* = 16
CSSA3	3.1 ± 0.7	0.43	*n* = 16
HY TME	1.9 ± 1.3	5.8	*n* = 16
HaCWD	2.6 ± 0.6	1.4	*n* = 16
263K scrapie	2.3 ± 0.5	2.4	*n* = 16
139H scrapie	2.8 ± 0.8	0.77	*n* = 16
ME7H scrapie	1.0 ± 0.0	50.0	*n* = 12

^*a*^ The data in Fig. 3 were used to determine PMCA conversion efficiency using two different calculations.

^*b*^ The uninfected samples represent individual technical replicates. All prion strain PMCA replicate numbers represent individual 10-fold serial dilutions of mouse or hamster prion strain-infected brain homogenate.

^*c*^ The average number of 10-fold serial dilutions of mouse or hamster brain homogenate that resulted in an extinction of prion conversion after one round of PMCA.

^*d*^ The lowest amount of microgram equivalents of mouse or hamster brain homogenate that resulted in detectable amplified PrP^Sc^ following one round of PMCA.

^*e*^ n.a., not applicable.

**Fig 4:**
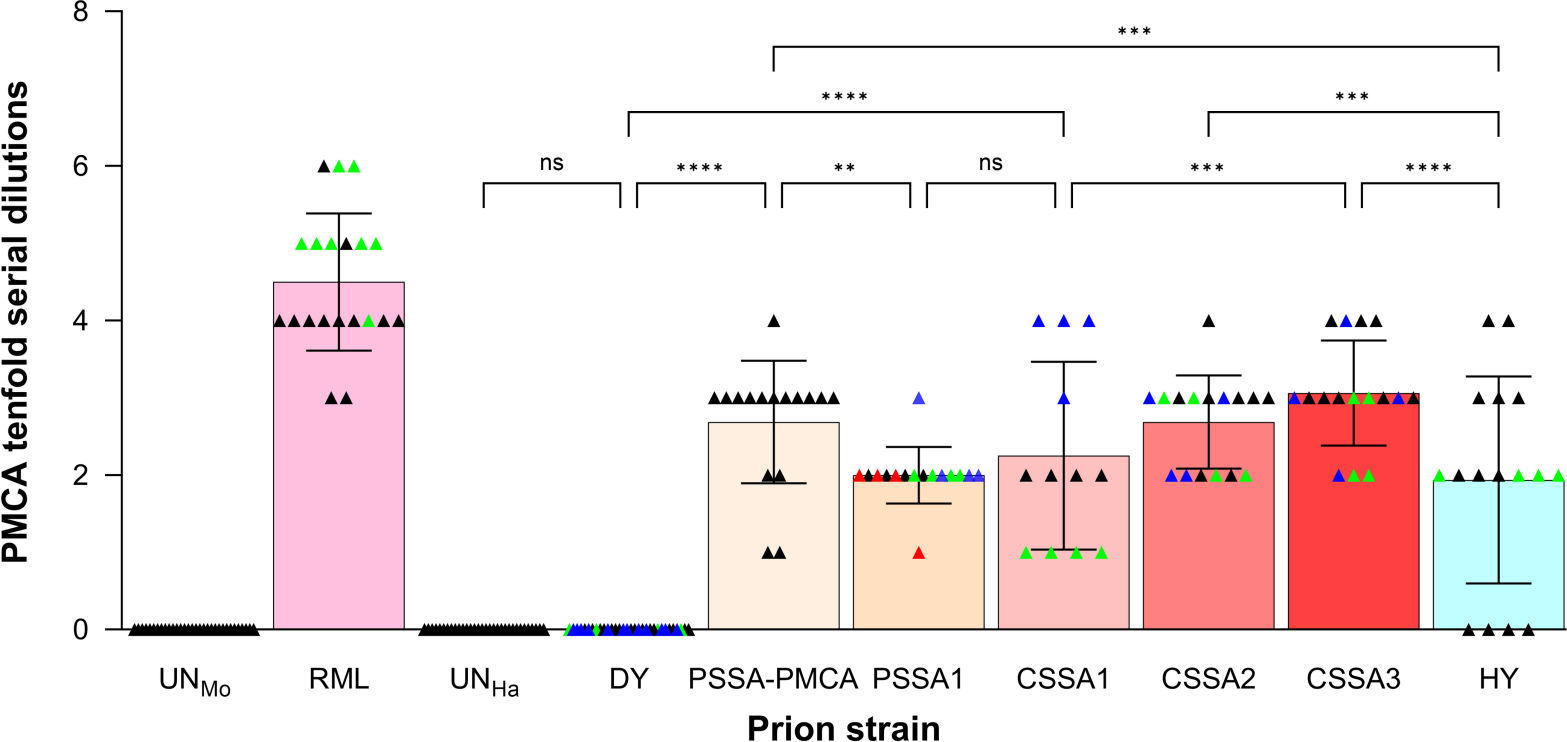
Hamster prion strains differ in interspecies PMCA conversion efficiency for mice. Quantification of PMCA conversion efficiency of 10-fold serial dilutions of hamster brain homogenate seeded into PMCA reactions using mouse brain homogenate as a template for conversion. Presented are all technical replicates for each prion sample, with means and standard deviations. Individual animals for each strain are represented by different colored triangles. ns, not significant (*P* > 0.05); **P < 0.01; ***P < 0.001, ****P < 0.0001. CSSA1, DY 4M CSSA hamster passage 1; CSSA2, DY 4M CSSA hamster passage 2; CSSA3, DY 4M CSSA hamster passage 3; DY, drowsy hamster-adapted TME; HY, hyper hamster-adapted TME; PSSA1, DY PSSA hamster passage 1; PSSA-PMCA, DY PSSA hamster PMCA round 4; RML, Rocky Mountain laboratory mouse-adapted scrapie; UN_Ha_, uninfected hamster; UN_Mo_, uninfected mouse.

To further expand upon this observation, all hamster minor strains (CSSA1, CSSA2, CSSA3, PSSA-PMCA, and PSSA1) isolated from DY TME were tested for the ability to convert mouse PrP^C^ to PrP^Sc^ using interspecies PMCA. We found that after one round of PMCA, all hamster minor strains resulted in conversion of mouse PrP^C^ into PrP^Sc^ (Fig. 3 and 4; Table 2). Overall, minor prion strains resulted in interspecies PMCA conversion where the dominant prion strain DY TME did not.

### Minor prion strains differ in interspecies PMCA conversion efficiency

The interspecies PMCA conversion efficiencies were compared between the minor prion strains and other well-characterized hamster-adapted prion strains. Ten-fold serial dilutions of brain homogenate from HY TME-, HaCWD-, 263K scrapie-, 139H scrapie-, and ME7H scrapie-infected hamster brain homogenate were seeded into uninfected mouse brain homogenate (Fig. 3, panel B) and used to determine interspecies PMCA conversion efficiency (Fig. 4; Fig. S3, panel B; Table 2). The interspecies PMCA conversion efficiencies were then compared between these known prion strains and the minor prion strains isolated from DY TME (Fig. 4; Fig. S3, panel B; Table 2). Significant differences in interspecies PMCA conversion efficiency were identified between the minor prion strains isolated from DY TME and other known hamster-adapted prion strains (Fig. 4; Fig. S3, panel B; Table 2). The lowest interspecies PMCA conversion efficiency identified was from ME7H (Fig. S3, panel B; Table 2), differing significantly (*P* < 0.0001) from all other hamster-adapted strains or minor prion strains tested. The highest interspecies PMCA conversion efficiency was from CSSA3, which differed significantly from 263K scrapie (*P* = 0.0009) and HaCWD (*P* = 0.0264) (Fig. 4; Fig. S3, panel B; Table 2). Interspecies PMCA conversion efficiencies for CSSA2, CSSA3, and PSSA1 differed significantly (*P* = 0.0009, *P* < 0.0001, and *P* = 0.0009, respectively) from HY TME (Fig. 4; Table 2). Overall, these data are consistent with the hypothesis that the efficiency of interspecies transmission is a strain-specific property.

### Minor prion strains convert in cell-based assay, where dominant strain does not convert

**Fig 5:**
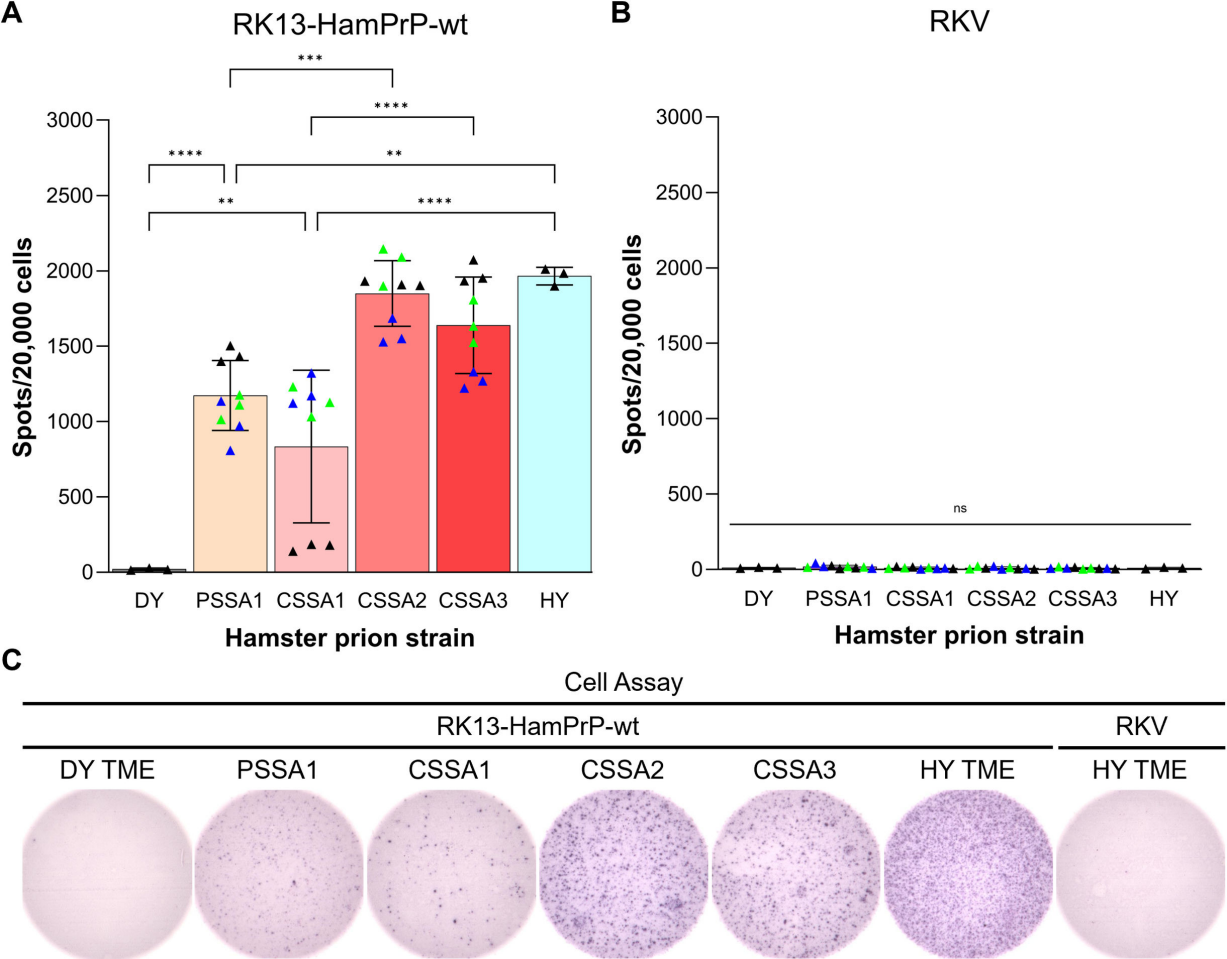
RK13-HamPrP cells are resistant to infection with DY TME but susceptible to minor DY strains. (**A**) RK13-HamPrP cells were incubated for 4 weeks in 96-well plates pre-coated with 1% brain homogenates from diseased hamsters inoculated with HY TME, DY TME, and minor strains derived from CSSA and PSSA treatments. A total of 20,000 cells were filtered onto ELISpot plates, digested with PK, denatured with guanidine thiocyanate, and probed with SHA31 PrP antibody. Mean ± standard deviation, counts of prion infected cells from three infected animals, where each of the animals were assayed in triplicate (CSSA and PSSA), or a single animal was assayed in triplicate (HY and DY). Statistical comparison was analyzed by one-way ANOVA with Tukey’s multiple comparison test. **P < 0.01; ***P < 0.001; ****P < 0.0001. (**B**) Parallel infections with RKV cells serve as negative controls. RKV: RK13 cells transfected with pIRESpuro3 vector do not express hamster PrP. Statistical comparison was analyzed by one-way ANOVA with Tukey’s multiple comparison test; ns, not significant (*P* > 0.05). (**C**) Representative wells of infections of RK13-HamPrP-wt cells with DY TME, PSSA1, CSSA1, CSSA2, CSSA3, and HY TME or RKV negative control cells with HY TME prions. Spots: PrP^Sc^ of prion infected cells were probed with SHA31 PrP antibody, followed by alkaline phosphatase-conjugated secondary antibody and visualized with NBT/BCIP substrate. ANOVA, analysis of variance; CSSA1, DY 4M CSSA hamster passage 1; CSSA2, DY 4M CSSA hamster passage 2; CSSA3, DY 4M CSSA hamster passage 3; PSSA1, DY PSSA hamster passage 1; RKV, RK-vector.

In rabbit kidney epithelial (RK13)-HamPrP-wt cells, the cell infection efficiencies of minor prion strains were compared to the dominant strain, DY TME (Fig. 5). DY TME failed to infect RK13-HamPrP-wt cells (Fig. 5, panel A). However, HY TME, PSSA1, CSSA1, CSSA2, and CSSA3 all infected RK13-HamPrP-wt cells (Fig. 5, panel A), differing significantly from DY TME in cell infection efficiency (*P* < 0.0001). HY TME had a significantly (*P* < 0.0001) higher average cell infection efficiency compared to CSSA1. Similarly, CSSA2 and CSSA3 had a significantly (*P* < 0.0001) higher cell infection efficiency than CSSA1, suggesting that the average cell infection efficiency of CSSA strains increased upon serial hamster passage (Fig. 5, panel A). Overall, minor prion strains emerging from CSSA and PSSA differed in cell infection efficiency from each other and from the dominant strain, DY TME.

## DISCUSSION

**Fig 6:**
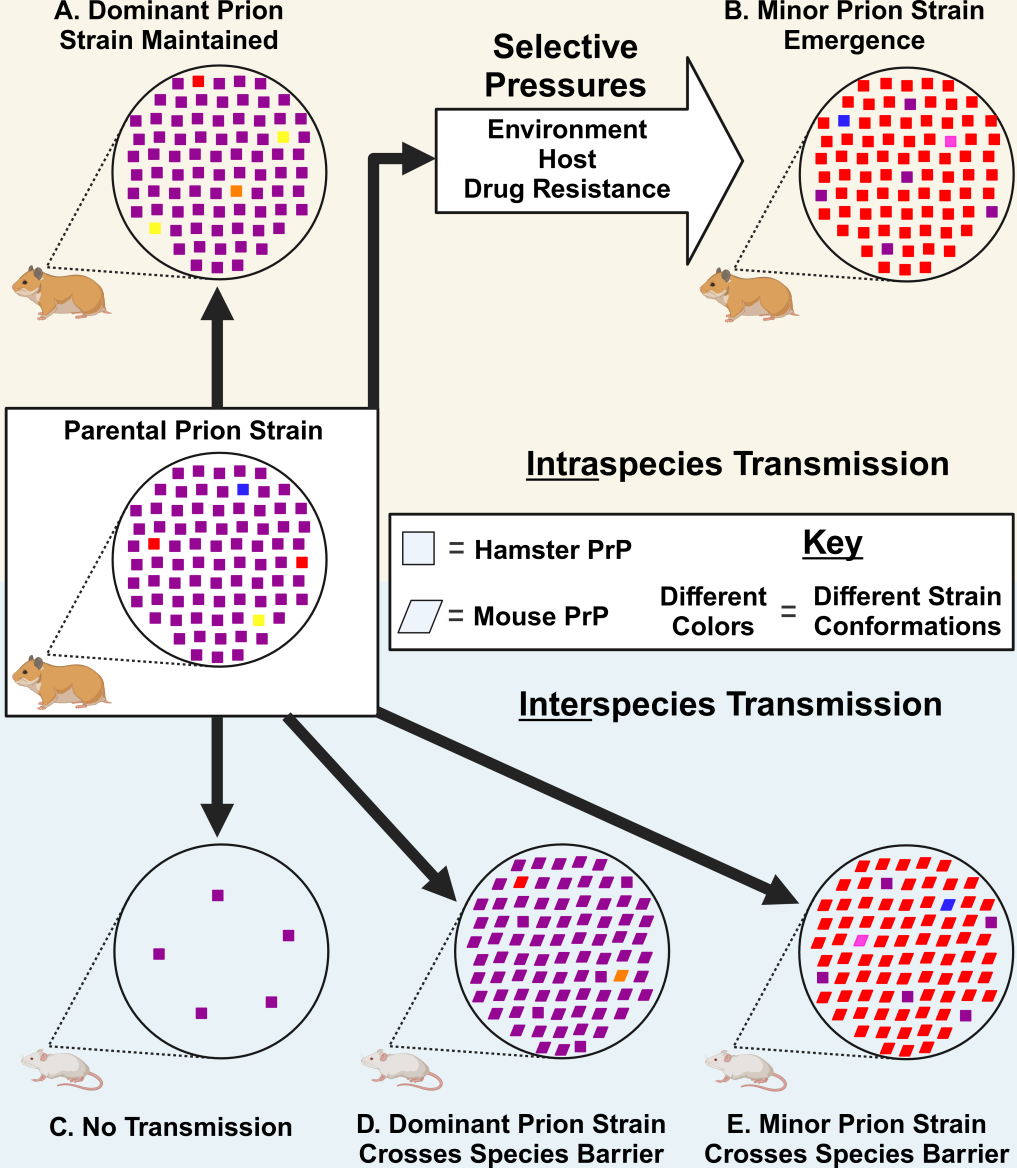
Theoretical model of prion transmission. (**A**) Serial intraspecies transmission of a prion strain most often results in the maintenance of the dominant prion strain. (**B**) Intraspecies transmission in the presence of a selective pressure (e.g., anti-prion drug) can result in the emergence of a pre-existing minor prion strain that has a replicative advantage compared to the parental dominant prion strain. Interspecies transmission can result in a (C) failure to establish infection, (**D**) the dominant prion strain resulting in the conversion of the new host PrP^C^ to PrP^Sc^, or (E) a minor strain component of the parental prion strain causing the conversion of PrP^C^ to PrP^Sc^ in the new host species. Created with BioRender.com.

The efficiency of interspecies prion transmission is influenced by both the host and prion strain. Differences in the amino acid sequence of PrP^C^ and PrP^Sc^, either within or between species, can greatly affect the efficiency of transmission (65, 66). Relatively less is known about the influence of the prion strain on efficiency of interspecies transmission, but evidence suggests that distinct prion strains in the same host species can have different efficiencies of establishing infection into a new host species (79–81). The Collinge and Clarke model of prion strain dynamics hypothesizes that the mutant spectra can enable prions to overcome the species barrier (82). As it is becoming increasingly clear that prion strains exist as a dominant strain and minor strains, we tested if the transmission potential of the minor prion strain component differs from the dominant prion strain, in this case DY TME. To directly test this hypothesis, we examined the ability of DY TME to convert mouse PrP^C^ to PrP^Sc^ and compared it to the pre-existing minor prion strains that we isolated previously from biologically cloned DY-infected hamster brain (63). We found that the dominant hamster prion strain, DY TME, was unable to convert mouse PrP^C^ to PrP^Sc^ after four serial rounds of PMCA (Fig. 3, panel A). Consistent with the Collinge and Clarke hypothesis, all minor prion strains examined isolated from biologically cloned DY TME using either CSSA or PSSA were successful at converting mouse PrP^C^ after one round of PMCA (Fig. 3 and 4; Table 2). Consecutive hamster passages of CSSA minor prion strains resulted in a decrease in the incubation period in hamsters (63). Here we determined that both the intraspecies and interspecies PMCA conversion efficiencies increased (Fig. 2 and 4), providing further evidence of prion strain evolution. Importantly, if a prion spillover event should occur, it should not be assumed that the interspecies transmission potential in the newly infected species is constant, as here we provide evidence that it can change with passage history. Finally, the uninfected negative control PMCA reactions did not convert PrP^C^ to PrP^Sc^ in either hamsters (*n* = 147; Table 1) or mice (*n* = 64; Table 2), suggesting that the interspecies transmission observed was not due to contamination or the spontaneous generation of prions. Overall, the PMCA conversion data are consistent with the hypothesis that minor prion strains can contribute to interspecies transmission (Fig. 6).

Minor prion strains can cross species barriers, allowing for a reinterpretation of previous work. Interspecies transmission of biologically cloned TME to hamsters resulted in the emergence of the HY and DY strains of hamster-adapted TME (83). It was hypothesized that mink PrP^Sc^ resulted in the production of either the HY or DY TME conformations of PrP^Sc^. As biologically cloned prions can contain a mutant spectra of minor prion strains, it is possible that the production of HY TME and DY TME is due to contributions of the mutant spectra present in the biologically cloned TME inoculum. The 263K strain of hamster-adapted scrapie was isolated from the SSBP/1 sheep scrapie pool that was passaged in mice and rats prior to transmission to hamsters (84). As 263K is poorly pathogenic in mice, it was difficult for the authors to provide a mechanism to explain this observation, and it was speculated that 263K could “replicate in mice but are completely non-pathogenic in this host,” or 263K arose by “host induced modification” (84). More recent findings that support the existence of pre-existing minor prion strains that can arise after biological cloning of a prion strain provide a plausible mechanism to explain the emergence of 263K. Additionally, failed attempts to cross the species barrier experimentally may be only assessing the transmission potential of the dominant prion strain population without considering the interspecies transmission potential of minor prion strains from the mutant spectra. Further work using methods to select for minor prion strains may enable a more complete assessment of the capacity of prion strains to overcome interspecies transmission barriers. This is particularly relevant when evaluating the potential of CWD strains to infect sympatric species, including humans.

Minor prion strains isolated from the mutant spectra of DY TME differed from HY TME and each other. The minor prion strains from the DY TME mutant spectra had significantly different intraspecies and interspecies PMCA conversion efficiencies compared to HY TME that was observed between several individual animals infected with each isolate (Fig. 2 and 4; Tables 1 and 2). This suggests that the measured differences in the minor prion strains were not due to HY contamination nor due to variability between animals or input PrP^Sc^. As prion strains are operationally defined, the biological importance of these observed differences is unclear. For example, the third hamster passage of the DY CSSA prion strain has a similar incubation period, clinical signs, PrP^Sc^ migration, and conformational stability compared to HY TME (63), but differs significantly in PMCA conversion efficiency from HY TME and from the first passage of DY CSSA (Fig. 2 and 4; Tables 1 and 2). We hypothesize that these highly related but not identical isolates may represent thermodynamic interconvertible prion substrains as proposed by Weissmann (61, 62). Therefore, if PrP^Sc^ conformation is the sole determinant of prion strain diversity. We would predict that the conformation of PrP^Sc^ from the mutant spectra substrains characterized here should also differ. Overall, this suggests that the diversity of prion quasispecies mutant spectra is greater than previously expected (Fig. 6).

## MATERIALS AND METHODS

### Animal bioassay and prion strains

Male Syrian golden hamsters (Harlan-Sprague-Dawley, Indianapolis, IN) were inoculated intracerebrally with 20 µL of a 1% wt/vol brain homogenate from a HY TME-infected hamster or PMCA reaction from either uninfected or DY-TME-infected PSSA products as described previously (63). The hamsters were observed three times a week for clinical signs of prion disease that include hyperexcitability and ataxia, and incubation periods were calculated as the difference in the number of days between onset of clinical signs and the inoculation date. After the hamsters were euthanized, brains and spleens were collected with tools dedicated specifically for each prion strain and hamster passage, and tools were decontaminated between collection of each animal by soaking them in bleach for 15 minutes. All tissues were placed immediately on dry ice, then stored at −80°C. Brains were homogenized in Dulbecco’s phosphate-buffered saline (DPBS; Corning, Manassas, VA) to 10% wt/vol using disposable conical tubes and a bead blaster (Omni International Incorporation, Kennesaw, GA).

### Western blot analysis

Western blot detection of PrP^Sc^ was performed as described previously (63). Brain homogenates were diluted to 5% wt/vol in DPBS containing Proteinase K (PK; final concentration of 23.75 µg/mL; Roche Diagnostics Corporation, Indianapolis, IN) and incubated at 37°C for 1 hour with shaking at 800 rpm. To stop PK digestion, samples were incubated for 10 minutes at 100°C with either a 1:1 ratio of 2× sample buffer for hamster PrP or a 2:1 ratio of 4× sample buffer for mouse PrP [4% or 8% wt/vol SDS (Thermo Fisher, Waltham, MA), respectively, 2% or 4% vol/vol β-mercaptoethanol (Sigma-Aldrich, Burlington, MA) respectively, 40% vol/vol glycerol (Thermo Fisher), and 0.004% wt/vol bromophenol blue (Sigma-Aldrich), in 0.5-M Tris buffer, pH 6.8]. Next, samples were size fractionated on 4%–12% Bis-Tris NuPAGE polyacrylamide gels (Invitrogen, Carlsbad, CA), then transferred to polyvinylidene difluoride membranes (Immobilon FL, Sigma-Aldrich) for an hour. Membranes were blocked for 30 minutes with 5% wt/vol non-fat dry milk in 0.05% vol/vol Tween Tris-buffered saline (Bio-Rad Laboratories, Hercules, CA). Hamster prion protein was detected using either mouse monoclonal anti-PrP antibody 12B2 (final concentration of 0.2 µg/mL; Wageningen Bioveterinary Research, Wageningen, the Netherlands) or 3F4 (final concentration of 0.1 µg/mL, Sigma-Aldrich) (85, 86). Alternatively, mouse prion protein was detected by mouse monoclonal anti-PrP antibody PRC7 (final concentration of 0.2 µg/mL), which recognizes mouse but not hamster PrP (78). Western blots were developed using Pierce SuperSignal West Femto maximum sensitivity substrate via manufacturer’s instructions (Pierce, Rockford, IL) and immediately captured on a Li-Cor Odyssey Fc Imager (Li-Cor, Lincoln, NE).

### PMCA

PMCA was performed as described previously (63). PMCA substrate was made from either uninfected hamster or mouse brains homogenized to 10% wt/vol in PMCA conversion buffer [DPBS (pH 7.4) containing 1% vol/vol Triton X-100 (Sigma-Aldrich), a complete protease inhibitor cocktail tablet (Sigma-Aldrich), 6-mM ethylenediaminetetraacetic acid (Sigma-Aldrich), 150-mM NaCl (Sigma-Aldrich), 100-µg/mL heparin (Sigma-Aldrich), and 0.05% wt/vol digitonin (Sigma-Aldrich)]. Samples were loaded into a Qsonica Q700MPX sonicator (Newtown, CT) and subjected to one round of PMCA at 37°C for 72 hours consisting of cycles of 1-second sonication for every 10-minute incubation. The sonicator output level varied from 37 to 40 with an output range of 230–280 W per sonicator cycle. After PMCA, PrP^Sc^ was detected by Western blot analysis as described above.

PMCA conversion efficiencies for both intraspecies and interspecies reactions were calculated using two different methods. First, endpoint 10-fold serial dilution values were calculated based upon the average number of 10-fold dilutions from the starting 10% wt/vol brain homogenate that resulted in a failure to detect PrP^Sc^ by Western blot after one round of PMCA. A higher number corresponds with greater efficiency. Second, microgram detection limit is the average minimum microgram equivalent of wet weight of brain that results in detection of PrP^Sc^ following one round of PMCA. Using this calculation of PMCA conversion efficiency, we found that a lower number corresponds with greater efficiency. PMCA conversion efficiencies were determined from each of three to four individual animals per strain or minor prion strain inoculated group. Differences between conversion efficiency for prion strains after one round of PMCA were analyzed statistically using one-way analysis of variance with uncorrected Fisher’s least significant difference (*P* < 0.05).

### RK13 cell infections

RK13 cells (ATCC, CCL-37) were transfected with empty pIRESpuro3 vector (Takara, Cat. # 631619) or recombinant pIRESpuro3-HamPrP expressing golden hamster Prnp gene (National Center for Biotechnology Information reference sequence: XM_013112401.2). These cell lines were referred to as RK-vector (RKV) and RK13-HamPrP, respectively. Both cell lines were cultured in Dulbecco’s Modified Eagle Medium containing 10% (vol/vol) heat-inactivated fetal bovine serum (Gibco, Cat. # 10082147), 1-µg/mL penicillin/100-U/mL streptomycin, and 1-µg/mL puromycin (Millipore Sigma, Cat. # P8833). Hamster prion cell assay was developed using the strategy previously described for cervid, ovine, and mouse prions (21, 87). Brain homogenates were generated by repeated passing through 18-, 22-, and 26-gauge blunt-end needles in phosphate-buffered saline (PBS) lacking calcium and magnesium ions. Wells of 96-well plates were coated with 100 µL, 1% of brain homogenates for 1 hour, then the brain homogenates were removed by aspiration and wells were washed twice with 150-µL PBS and air-dried. Twenty thousand RK13-HamPrP or RKV cells were added to each of the pre-coated wells in a volume of 100-µL culture medium. Cells were maintained for 4 weeks, and 150-µL cell culture medium was changed very 5 days. Four weeks post infection, cells were trypsinized, and 20,000 cells were filtered onto ELISpot (Millipore, Cat. # MSIPN4W50) plates, treated with proteinase K (5 µg/mL), followed by 3-M guanidine thiocyanate denaturation, immunoblotted with SHA31 (Cayman Chemical, Cat. # 11866) primary antibody, and AP-conjugated goat anti-mouse IgG secondary antibody. The proteinase K-resistant PrP^Sc^ signals were visualized with nitro blue tetrazolium/5-bromo-4-chloro-3-indolyl phosphate (NBT/BCIP) solution (Millipore Sigma, Cat. # 11681451001), and after being air-dried, the plates were scanned and analyzed using ImmunoSpot analyzer, S6UNV2 (ImmunoSpot, Cleveland, OH). Cell infection efficiency is defined as the number of PrP^Sc^ spots per 20,000 cells (87) with a greater number of spots equating to greater infection efficiency.

## SUPPLEMENTAL MATERIAL

Supplemental figures mbio.02721-24-s0001.docx Figures S1 through S4.

ASM does not own the copyrights to Supplemental Material that may be linked to, or accessed through, an article. The authors have granted ASM a non-exclusive, world-wide license to publish the Supplemental Material files. Please contact the corresponding author directly for reuse.

## References

[1] Prusiner SB. 1982. Novel proteinaceous infectious particles cause scrapie. Science 216:136–144. doi:10.1126/science.6801762 6801762

[2] Kong Q, Bessen RA. 2017. Prion diseases, p 517–531. In Ikezu T, Gendelman HE (ed), Neuroimmune pharmacology. Springer international publishing, Switzerland.

[3] Ironside JW, Ritchie DL, Head MW. 2018. Prion diseases, p 393–403. In Kovacs GG, Alafuzoff I (ed), Neuropathology. Elsevier B.V.

[4] Orge L, Lima C, Machado C, Tavares P, Mendonça P, Carvalho P, Silva J, Pinto M de L, Bastos E, Pereira JC, Gonçalves-Anjo N, Gama A, Esteves A, Alves A, Matos AC, Seixas F, Silva F, Pires I, Figueira L, Vieira-Pinto M, Sargo R, Pires MDA. 2021. Neuropathology of animal prion diseases. Biomolecules 11:466. doi:10.3390/biom11030466 33801117 PMC8004141

[5] Marsh RF, Bessen RA, Lehmann S, Hartsough GR. 1991. Epidemiological and experimental studies on a new incident of transmissible mink encephalopathy. J Gen Virol 72 (Pt 3):589–594. doi:10.1099/0022-1317-72-3-589 1826023

[6] Baron T, Bencsik A, Biacabe A-G, Morignat E, Bessen RA. 2007. Phenotypic similarity of transmissible mink encephalopathy in cattle and L-type bovine spongiform encephalopathy in a mouse model. Emerg Infect Dis 13:1887–1894. doi:10.3201/eid1312.070635 18258040 PMC2876762

[7] Wilesmith JW, Wells GA, Cranwell MP, Ryan JB. 1988. Bovine spongiform encephalopathy: epidemiological studies. Vet Rec 123:638–644. 3218047

[8] Kirkwood JK, Wells GA, Wilesmith JW, Cunningham AA, Jackson SI. 1990. Spongiform encephalopathy in an arabian oryx (Oryx leucoryx) and a greater kudu (Tragelaphus strepsiceros). Vet Rec 127:418–420. 2264242

[9] Fraser H, Pearson GR, McConnell I, Bruce ME, Wyatt JM, Gruffydd-Jones TJ. 1994. Transmission of feline spongiform encephalopathy to mice. Vet Rec 134:449. doi:10.1136/vr.134.17.449 8048218

[10] Lezmi S, Bencsik A, Baron T. 2006. PET-blot analysis contributes to BSE strain recognition in C57Bl/6 mice. J Histochem Cytochem 54:1087–1094. doi:10.1369/jhc.5A6892.2006 16735593 PMC3957803

[11] Bons N, Mestre-Frances N, Belli P, Cathala F, Gajdusek DC, Brown P. 1999. Natural and experimental oral infection of nonhuman primates by bovine spongiform encephalopathy agents. Proc Natl Acad Sci U S A 96:4046–4051. doi:10.1073/pnas.96.7.4046 10097160 PMC22417

[12] Collinge J, Sidle KC, Meads J, Ironside J, Hill AF. 1996. Molecular analysis of prion strain variation and the aetiology of 'new variant' CJD. Nat New Biol 383:685–690. doi:10.1038/383685a0 8878476

[13] Will RG, Ironside JW, Zeidler M, Cousens SN, Estibeiro K, Alperovitch A, Poser S, Pocchiari M, Hofman A, Smith PG. 1996. A new variant of Creutzfeldt-Jakob disease in the UK. Lancet 347:921–925. doi:10.1016/s0140-6736(96)91412-9 8598754

[14] Bruce ME, Will RG, Ironside JW, McConnell I, Drummond D, Suttie A, McCardle L, Chree A, Hope J, Birkett C, Cousens S, Fraser H, Bostock CJ. 1997. Transmissions to mice indicate that 'new variant' CJD is caused by the BSE agent. Nat New Biol 389:498–501. doi:10.1038/39057 9333239

[15] Hill AF, Desbruslais M, Joiner S, Sidle KC, Gowland I, Collinge J, Doey LJ, Lantos P. 1997. The same prion strain causes vCJD and BSE. Nature New Biol 389:448–450, doi:10.1038/38925 9333232

[16] Béringue V, Herzog L, Reine F, Le Dur A, Casalone C, Vilotte J-L, Laude H. 2008. Transmission of atypical bovine prions to mice transgenic for human prion protein. Emerg Infect Dis 14:1898–1901. doi:10.3201/eid1412.080941 19046515 PMC2634647

[17] Comoy EE, Mikol J, Ruchoux M-M, Durand V, Luccantoni-Freire S, Dehen C, Correia E, Casalone C, Richt JA, Greenlee JJ, Torres JM, Brown P, Deslys J-P. 2013. Evaluation of the zoonotic potential of transmissible mink encephalopathy. Pathogens 2:520–532. doi:10.3390/pathogens2030520 25437205 PMC4235697

[18] Padilla D, Béringue V, Espinosa JC, Andreoletti O, Jaumain E, Reine F, Herzog L, Gutierrez-Adan A, Pintado B, Laude H, Torres JM. 2011. Sheep and goat BSE propagate more efficiently than cattle BSE in human PrP transgenic mice. PLoS Pathog 7:e1001319. doi:10.1371/journal.ppat.1001319 21445238 PMC3060172

[19] Pirisinu L, Tran L, Chiappini B, Vanni I, Di Bari MA, Vaccari G, Vikøren T, Madslien KI, Våge J, Spraker T, Mitchell G, Balachandran A, Baron T, Casalone C, Rolandsen CM, Røed KH, Agrimi U, Nonno R, Benestad SL. 2018. Novel type of chronic wasting disease detected in moose (Alces alces), Norway. Emerg Infect Dis 24:2210–2218. doi:10.3201/eid2412.180702 30457526 PMC6256397

[20] Nonno R, Di Bari MA, Pirisinu L, D’Agostino C, Vanni I, Chiappini B, Marcon S, Riccardi G, Tran L, Vikøren T, Våge J, Madslien K, Mitchell G, Telling GC, Benestad SL, Agrimi U. 2020. Studies in bank voles reveal strain differences between chronic wasting disease prions from Norway and North America. Proc Natl Acad Sci U S A 117:31417–31426. doi:10.1073/pnas.2013237117 33229531 PMC7733848

[21] Bian J, Kim S, Kane SJ, Crowell J, Sun JL, Christiansen J, Saijo E, Moreno JA, DiLisio J, Burnett E, Pritzkow S, Gorski D, Soto C, Kreeger TJ, Balachandran A, Mitchell G, Miller MW, Nonno R, Vikøren T, Våge J, Madslien K, Tran L, Vuong TT, Benestad SL, Telling GC. 2021. Adaptive selection of a prion strain conformer corresponding to established North American CWD during propagation of novel emergent Norwegian strains in mice expressing elk or deer prion protein. PLoS Pathog 17:e1009748. doi:10.1371/journal.ppat.1009748 34310663 PMC8341702

[22] Pritzkow S, Gorski D, Ramirez F, Telling GC, Benestad SL, Soto C. 2022. North American and Norwegian chronic wasting disease prions exhibit different potential for interspecies transmission and zoonotic risk. J Infect Dis 225:542–551. doi:10.1093/infdis/jiab385 34302479 PMC8807243

[23] Sola D, Tran L, Våge J, Madslien K, Vuong TT, Korpenfelt SL, Ågren EO, Averhed G, Nöremark M, Sörén K, Isaksson M, Acín C, Badiola JJ, Gavier-Widén D, Benestad SL. 2023. Heterogeneity of pathological prion protein accumulation in the brain of moose (Alces alces) from Norway, Sweden and Finland with chronic wasting disease. Vet Res 54:74. doi:10.1186/s13567-023-01208-3 37684668 PMC10492377

[24] Sun JL, Kim S, Crowell J, Webster BK, Raisley EK, Lowe DC, Bian J, Korpenfelt S-L, Benestad SL, Telling GC. 2023. Novel prion strain as cause of chronic wasting disease in a moose, Finland. Emerg Infect Dis 29:323–332. doi:10.3201/eid2902.220882 36692340 PMC9881765

[25] Bartz JC. 2016. Prion strain diversity. Cold Spring Harb Perspect Med 6:12. doi:10.1101/cshperspect.a024349 PMC5131755 27908925

[26] Bessen RA, Marsh RF. 1992. Biochemical and physical properties of the prion protein from two strains of the transmissible mink encephalopathy agent. J Virol 66:2096–2101. doi:10.1128/JVI.66.4.2096-2101.1992 1347795 PMC289000

[27] Bessen RA, Marsh RF. 1992. Identification of two biologically distinct strains of transmissible mink encephalopathy in hamsters. J Gen Virol 73 ( Pt 2):329–334. doi:10.1099/0022-1317-73-2-329 1531675

[28] Dickinson AG. 1976. Scrapie in sheep and goats, p 209–241. In Kimberlin R (ed), Slow virus diseases of animals and man. North-Holland Publishing Company, Amsterdam.

[29] Duque Velásquez C, Kim C, Herbst A, Daude N, Garza MC, Wille H, Aiken J, McKenzie D. 2015. Deer prion proteins modulate the emergence and adaptation of chronic wasting disease strains. J Virol 89:12362–12373. doi:10.1128/JVI.02010-15 26423950 PMC4665243

[30] Bessen RA, Marsh RF. 1994. Distinct PrP properties suggest the molecular basis of strain variation in transmissible mink encephalopathy. J Virol 68:7859–7868. doi:10.1128/JVI.68.12.7859-7868.1994 7966576 PMC237248

[31] Parchi P, Castellani R, Capellari S, Ghetti B, Young K, Chen SG, Farlow M, Dickson DW, Sima AA, Trojanowski JQ, Petersen RB, Gambetti P. 1996. Molecular basis of phenotypic variability in sporadic Creutzfeldt-Jakob disease. Ann Neurol 39:767–778. doi:10.1002/ana.410390613 8651649

[32] Castilla J, Gonzalez-Romero D, Saá P, Morales R, De Castro J, Soto C. 2008. Crossing the species barrier by PrP(Sc) replication in vitro generates unique infectious prions. Cell 134:757–768. doi:10.1016/j.cell.2008.07.030 18775309 PMC2740631

[33] Deleault AM, Deleault NR, Harris BT, Rees JR, Supattapone S. 2008. The effects of prion protein proteolysis and disaggregation on the strain properties of hamster scrapie. J Gen Virol 89:2642–2650. doi:10.1099/vir.0.2008/002303-0 18796735 PMC2675185

[34] Ayers JI, Schutt CR, Shikiya RA, Aguzzi A, Kincaid AE, Bartz JC. 2011. The strain-encoded relationship between PrP replication, stability and processing in neurons is predictive of the incubation period of disease. PLoS Pathog 7:e1001317. doi:10.1371/journal.ppat.1001317 21437239 PMC3060105

[35] Hallinan GI, Ozcan KA, Hoq MR, Cracco L, Vago FS, Bharath SR, Li D, Jacobsen M, Doud EH, Mosley AL, Fernandez A, Garringer HJ, Jiang W, Ghetti B, Vidal R. 2022. Cryo-EM structures of prion protein filaments from Gerstmann-Sträussler-Scheinker disease. Acta Neuropathol 144:509–520. doi:10.1007/s00401-022-02461-0 35819518 PMC9381446

[36] Hoyt F, Alam P, Artikis E, Schwartz CL, Hughson AG, Race B, Baune C, Raymond GJ, Baron GS, Kraus A, Caughey B. 2022. Cryo-EM of prion strains from the same genotype of host identifies conformational determinants. PLoS Pathog 18:e1010947. doi:10.1371/journal.ppat.1010947 36342968 PMC9671466

[37] Manka SW, Zhang W, Wenborn A, Betts J, Joiner S, Saibil HR, Collinge J, Wadsworth JDF. 2022. 2.7 Å cryo-EM structure of ex vivo RML prion fibrils. Nat Commun 13:4004. doi:10.1038/s41467-022-30457-7 35831275 PMC9279362

[38] Manka SW, Wenborn A, Betts J, Joiner S, Saibil HR, Collinge J, Wadsworth JDF. 2023. A structural basis for prion strain diversity. Nat Chem Biol 19:607–613. doi:10.1038/s41589-022-01229-7 36646960 PMC10154210

[39] Parchi P, Giese A, Capellari S, Brown P, Schulz-Schaeffer W, Windl O, Zerr I, Budka H, Kopp N, Piccardo P, Poser S, Rojiani A, Streichemberger N, Julien J, Vital C, Ghetti B, Gambetti P, Kretzschmar H. 1999. Classification of sporadic Creutzfeldt-Jakob disease based on molecular and phenotypic analysis of 300 subjects. Ann Neurol 46:224–233. 10443888

[40] Puoti G, Giaccone G, Rossi G, Canciani B, Bugiani O, Tagliavini F. 1999. Sporadic Creutzfeldt-Jakob disease: co-occurrence of different types of PrP(Sc) in the same brain. Neurology (ECronicon) 53:2173–2176. doi:10.1212/wnl.53.9.2173 10599800

[41] Cali I, Castellani R, Alshekhlee A, Cohen Y, Blevins J, Yuan J, Langeveld JPM, Parchi P, Safar JG, Zou W-Q, Gambetti P. 2009. Co-existence of scrapie prion protein types 1 and 2 in sporadic Creutzfeldt-Jakob disease: its effect on the phenotype and prion-type characteristics. Brain (Bacau) 132:2643–2658. doi:10.1093/brain/awp196 PMC2766234 19734292

[42] Parchi P, Saverioni D. 2012. Molecular pathology, classification, and diagnosis of sporadic human prion disease variants. Folia Neuropathol 50:20–45. 22505361

[43] Thackray AM, Lockey R, Beck KE, Spiropoulos J, Bujdoso R. 2012. Evidence for co-infection of ovine prion strains in classical scrapie isolates. J Comp Pathol 147:316–329. doi:10.1016/j.jcpa.2012.01.009 22522075

[44] Nonno R, Marin-Moreno A, Carlos Espinosa J, Fast C, Van Keulen L, Spiropoulos J, Lantier I, Andreoletti O, Pirisinu L, Di Bari MA, Aguilar-Calvo P, Sklaviadis T, Papasavva-Stylianou P, Acutis PL, Acin C, Bossers A, Jacobs JG, Vaccari G, D’Agostino C, Chiappini B, Lantier F, Groschup MH, Agrimi U, Maria Torres J, Langeveld JPM. 2020. Characterization of goat prions demonstrates geographical variation of scrapie strains in Europe and reveals the composite nature of prion strains. Sci Rep 10:19. doi:10.1038/s41598-019-57005-6 31913327 PMC6949283

[45] Marín-Moreno A, Aguilar-Calvo P, Espinosa JC, Zamora-Ceballos M, Pitarch JL, González L, Fernández-Borges N, Orge L, Andréoletti O, Nonno R, Torres JM. 2021. Classical scrapie in small ruminants is caused by at least four different prion strains. Vet Res 52:57. doi:10.1186/s13567-021-00929-7 33858518 PMC8048364

[46] Dickinson AG, Fraser H, Meikle VM, Outram GW. 1972. Competition between different scrapie agents in mice. Nat New Biol 237:244–245. doi:10.1038/newbio237244a0 4624846

[47] Dickinson AG, Fraser H, McConnell I, Outram GW, Sales DI, Taylor DM. 1975. Extraneural competition between different scrapie agents leading to loss of infectivity. Nature New Biol 253:556. doi:10.1038/253556a0 804143

[48] Bruce ME, Dickinson AG. 1979. Biological stability of different classes of scrapie agent, p 71–86. In Prusiner SB, Hadlow WJ (ed), Slow transmissible diseases of the nervous system volume 2 pathogenesis, immunology, virology, and molecular biology of the spongiform encephalopathies. Academic Press, New York.

[49] Dickinson AG, Outram GW. 1979. The scrapie replication-site hypothesis and its implications for pathogenesis, p 13–31. In Prusiner SB, Hadlow WJ (ed), Slow transmissible diseases of the nervous system. Vol. 2. Academic Press, New York.

[50] Bartz JC, Aiken JM, Bessen RA. 2004. Delay in onset of prion disease for the HY strain of transmissible mink encephalopathy as a result of prior peripheral inoculation with the replication-deficient DY strain. J Gen Virol 85:265–273. doi:10.1099/vir.0.19394-0 14718642

[51] Bartz JC, Kramer ML, Sheehan MH, Hutter JAL, Ayers JI, Bessen RA, Kincaid AE. 2007. Prion interference is due to a reduction in strain-specific PrPSc levels. J Virol 81:689–697. doi:10.1128/JVI.01751-06 17079313 PMC1797475

[52] Shikiya RA, Ayers JI, Schutt CR, Kincaid AE, Bartz JC. 2010. Coinfecting prion strains compete for a limiting cellular resource. J Virol 84:5706–5714. doi:10.1128/JVI.00243-10 20237082 PMC2876617

[53] Holec SAM, Yuan Q, Bartz JC. 2019. Alteration of prion strain emergence by nonhost factors. mSphere 4:e00630-19. doi:10.1128/mSphere.00630-19 31597719 PMC6796975

[54] Ghaemmaghami S, Ahn M, Lessard P, Giles K, Legname G, DeArmond SJ, Prusiner SB. 2009. Continuous quinacrine treatment results in the formation of drug-resistant prions. PLoS Pathog 5:e1000673. doi:10.1371/journal.ppat.1000673 19956709 PMC2777304

[55] Li J, Mahal SP, Demczyk CA, Weissmann C. 2011. Mutability of prions. EMBO Rep 12:1243–1250. doi:10.1038/embor.2011.191 21997293 PMC3245691

[56] Berry D, Giles K, Oehler A, Bhardwaj S, DeArmond SJ, Prusiner SB. 2015. Use of a 2-aminothiazole to treat chronic wasting disease in transgenic mice. J Infect Dis 212 Suppl 1:S17–S25. doi:10.1093/infdis/jiu656 26116725 PMC4551108

[57] Burke CM, Mark KMK, Kun J, Beauchemin KS, Supattapone S. 2020. Emergence of prions selectively resistant to combination drug therapy. PLoS Pathog 16:e1008581. doi:10.1371/journal.ppat.1008581 32421750 PMC7259791

[58] Beauchemin KS, Rees JR, Supattapone S. 2021. Alternating anti-prion regimens reduce combination drug resistance but do not further extend survival in scrapie-infected mice. J Gen Virol 102:12. doi:10.1099/jgv.0.001705 PMC8744272 34904943

[59] Li J, Browning S, Mahal SP, Oelschlegel AM, Weissmann C. 2010. Darwinian evolution of prions in cell culture. Science 327:869–872. doi:10.1126/science.1183218 20044542 PMC2848070

[60] Oelschlegel AM, Weissmann C. 2013. Acquisition of drug resistance and dependence by prions. PLoS Pathog 9:e1003158. doi:10.1371/journal.ppat.1003158 23408888 PMC3567182

[61] Weissmann C, Li J, Mahal SP, Browning S. 2011. Prions on the move. EMBO Rep 12:1109–1117. doi:10.1038/embor.2011.192 21997298 PMC3207107

[62] Weissmann C. 2012. Mutation and selection of prions. PLoS Pathog 8:e1002582. doi:10.1371/journal.ppat.1002582 22479179 PMC3315487

[63] Gunnels T, Shikiya RA, York TC, Block AJ, Bartz JC. 2023. Evidence for preexisting prion substrain diversity in a biologically cloned prion strain. PLoS Pathog 19:e1011632. doi:10.1371/journal.ppat.1011632 37669293 PMC10503715

[64] Outram GW. 1976. The pathogenesis of scrapie in mice, p 325–357. In Kimberlin RH (ed), Slow virus diseases of animals and man. Amsterdam, North-Holland Publ.

[65] Dickinson AG, Fraser H. 1979. An assessment of the genetics of scrapie in sheep and mice, p 367–385. In Prusiner SB, Hadlow WJ (ed), Slow transmissible diseases of the nervous system volume 1 clinical, epidemiological, genetic, and pathological aspects of the spongiform encephalopathies. Academic Press, Inc, New York, New York.

[66] Scott M, Groth D, Foster D, Torchia M, Yang SL, DeArmond SJ, Prusiner SB. 1993. Propagation of prions with artificial properties in transgenic mice expressing chimeric PrP genes. Cell 73:979–988. doi:10.1016/0092-8674(93)90275-u 8098995

[67] Gajdusek DC, Gibbs CJ. 1975. Slow virus infections of the nervous system and the laboratories of slow, latent and temperate virus infections, p 113–135. In Tower DB (ed), The clinical neurosciences. Vol. Raven Press.

[68] Kimberlin RH, Walker C. 1977. Characteristics of a short incubation model of scrapie in the golden hamster. J Gen Virol 34:295–304. doi:10.1099/0022-1317-34-2-295 402439

[69] Bartz JC, Marsh RF, McKenzie DI, Aiken JM. 1998. The host range of chronic wasting disease is altered on passage in ferrets. Virology (Auckl) 251:297–301. doi:10.1006/viro.1998.9427 9837794

[70] Bian J, Khaychuk V, Angers RC, Fernández-Borges N, Vidal E, Meyerett-Reid C, Kim S, Calvi CL, Bartz JC, Hoover EA, Agrimi U, Richt JA, Castilla J, Telling GC. 2017. Prion replication without host adaptation during interspecies transmissions. Proc Natl Acad Sci U S A 114:1141–1146. doi:10.1073/pnas.1611891114 28096357 PMC5293081

[71] Priola SA, Chesebro B. 1995. A single hamster PrP amino acid blocks conversion to protease-resistant PrP in scrapie-infected mouse neuroblastoma cells. J Virol 69:7754–7758. doi:10.1128/JVI.69.12.7754-7758.1995 7494285 PMC189717

[72] Telling GC, Haga T, Torchia M, Tremblay P, DeArmond SJ, Prusiner SB. 1996. Interactions between wild-type and mutant prion proteins modulate neurodegeneration in transgenic mice. Genes Dev 10:1736–1750. doi:10.1101/gad.10.14.1736 8698234

[73] Priola SA, Chabry J, Chan K. 2001. Efficient conversion of normal prion protein (PrP) by abnormal hamster PrP is determined by homology at amino acid residue 155. J Virol 75:4673–4680. doi:10.1128/JVI.75.10.4673-4680.2001 11312338 PMC114221

[74] Arifin MI, Kaczmarczyk L, Zeng D, Hannaoui S, Lee C, Chang SC, Mitchell G, McKenzie D, Beekes M, Jackson W, Gilch S. 2023. Heterozygosity for cervid S138N polymorphism results in subclinical CWD in gene-targeted mice and progressive inhibition of prion conversion. Proc Natl Acad Sci U S A 120:e2221060120. doi:10.1073/pnas.2221060120 37014866 PMC10104538

[75] Horiuchi M, Priola SA, Chabry J, Caughey B. 2000. Interactions between heterologous forms of prion protein: binding, inhibition of conversion, and species barriers. Proc Natl Acad Sci U S A 97:5836–5841. doi:10.1073/pnas.110523897 10811921 PMC18520

[76] Kraus A, Hoyt F, Schwartz CL, Hansen B, Artikis E, Hughson AG, Raymond GJ, Race B, Baron GS, Caughey B. 2021. High-resolution structure and strain comparison of infectious mammalian prions. Mol Cell 81:4540–4551. doi:10.1016/j.molcel.2021.08.011 34433091

[77] Artikis E, Kraus A, Caughey B. 2022. Structural biology of ex vivo mammalian prions. J Biol Chem 298:102181. doi:10.1016/j.jbc.2022.102181 35752366 PMC9293645

[78] Kang H-E, Weng CC, Saijo E, Saylor V, Bian J, Kim S, Ramos L, Angers R, Langenfeld K, Khaychuk V, Calvi C, Bartz J, Hunter N, Telling GC. 2012. Characterization of conformation-dependent prion protein epitopes. J Biol Chem 287:37219–37232. doi:10.1074/jbc.M112.395921 22948149 PMC3481321

[79] Nonno R, Di Bari MA, Cardone F, Vaccari G, Fazzi P, Dell’Omo G, Cartoni C, Ingrosso L, Boyle A, Galeno R, Sbriccoli M, Lipp H-P, Bruce M, Pocchiari M, Agrimi U. 2006. Efficient transmission and characterization of Creutzfeldt-Jakob disease strains in bank voles. PLoS Pathog 2:e12. doi:10.1371/journal.ppat.0020012 16518470 PMC1383487

[80] Bruce ME, Nonno R, Foster J, Goldmann W, Di Bari M, Esposito E, Benestad SL, Hunter N, Agrimi U. 2007. Nor98-like sheep scrapie in the United Kingdom in 1989. Vet Rec 160:665–666. doi:10.1136/vr.160.19.665 17496276

[81] Moore SJ, Smith JD, Richt JA, Greenlee JJ. 2019. Raccoons accumulate PrP^Sc^ after intracranial inoculation of the agents of chronic wasting disease or transmissible mink encephalopathy but not atypical scrapie. J Vet Diagn Invest 31:200–209. doi:10.1177/1040638718825290 30694116 PMC6838818

[82] Collinge J, Clarke AR. 2007. A general model of prion strains and their pathogenicity. Science 318:930–936. doi:10.1126/science.1138718 17991853

[83] Bartz JC, Bessen RA, McKenzie D, Marsh RF, Aiken JM. 2000. Adaptation and selection of prion protein strain conformations following interspecies transmission of transmissible mink encephalopathy. J Virol 74:5542–5547. doi:10.1128/jvi.74.12.5542-5547.2000 10823860 PMC112040

[84] Kimberlin RH, Walker CA. 1978. Evidence that the transmission of one source of scrapie agent to hamsters involves separation of agent strains from a mixture. J Gen Virol 39:487–496. doi:10.1099/0022-1317-39-3-487 96212

[85] Jeffrey M, Martin S, González L, Foster J, Langeveld JPM, van Zijderveld FG, Grassi J, Hunter N. 2006. Immunohistochemical features of PrP(d) accumulation in natural and experimental goat transmissible spongiform encephalopathies. J Comp Pathol 134:171–181. doi:10.1016/j.jcpa.2005.10.003 16542672

[86] Kascsak RJ, Rubenstein R, Merz PA, Tonna-DeMasi M, Fersko R, Carp RI, Wisniewski HM, Diringer H. 1987. Mouse polyclonal and monoclonal antibody to scrapie-associated fibril proteins. J Virol 61:3688–3693. doi:10.1128/JVI.61.12.3688-3693.1987 2446004 PMC255980

[87] Bian J, Napier D, Khaychuck V, Angers R, Graham C, Telling G. 2010. Cell-based quantification of chronic wasting disease prions. J Virol 84:8322–8326. doi:10.1128/JVI.00633-10 20519392 PMC2916541

