# Supplementary material for: Minor prion substrains overcome transmission barriers
Source: mBio. 2024 Oct 23;15(11):e02721-24. doi: 10.1128/mbio.02721-24 (PMC11559082; doi:10.1128/mbio.02721-24)
Supplement: Supplemental figures — Figures S1 through S4. [file mbio.02721-24-s0001.docx]

**Supplemental figures**


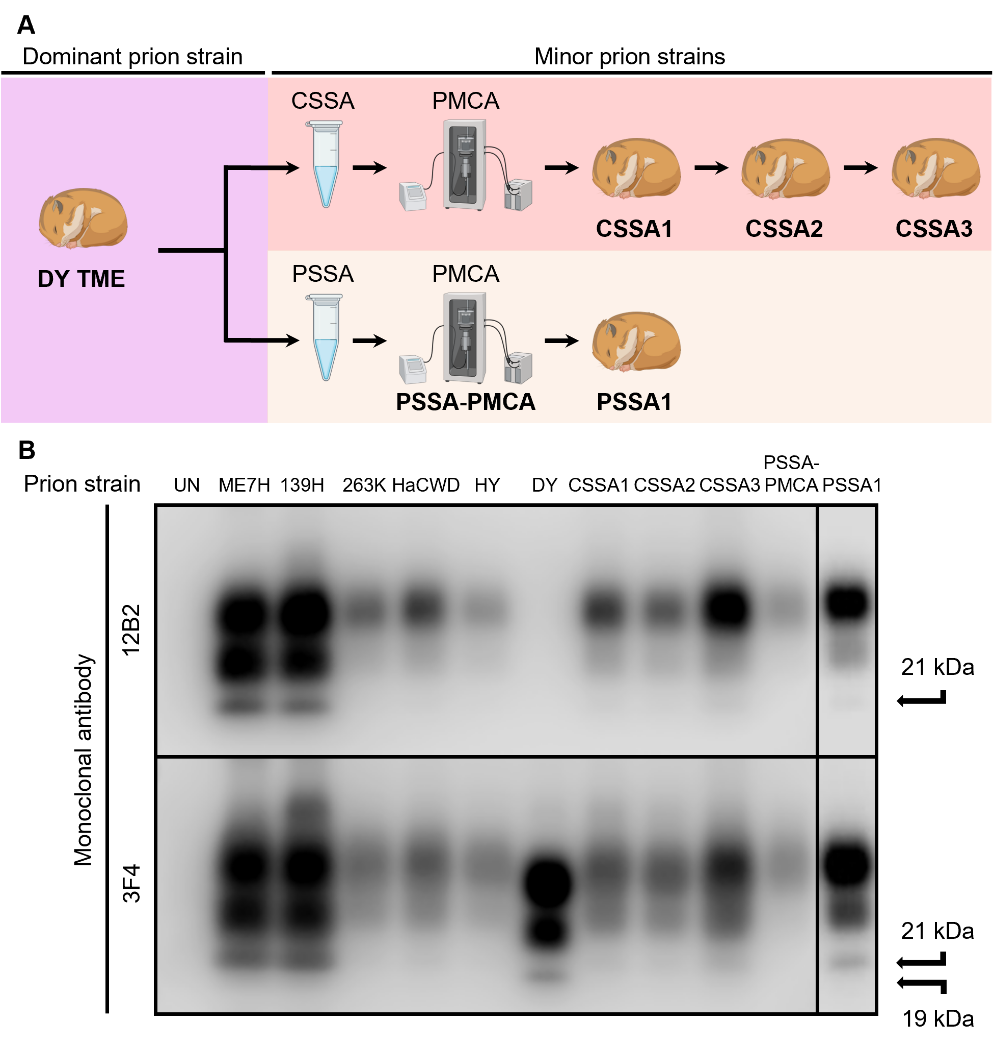


**Supplemental Figure 1. Passage history and Western blot analysis of PrP^Sc^ from hamster-adapted strains and minor strains isolated from DY TME using CSSA or PSSA.** A) Passage history of minor strains isolated from biologically cloned DY TME using CSSA or PSSA. Bold text indicates the designations used in the manuscript. B) Antibody affinity and electrophoretic migration of PrP^Sc^ from prion strains and minor strains that were first probed with anti-mouse primary antibody 12B2 and then reprobed with anti-mouse primary antibody 3F4. *Abbreviations:* UN, uninfected hamster; HY, hyper hamster-adapted TME; DY, drowsy hamster-adapted TME; CSSA1, DY 4M CSSA hamster passage 1; CSSA2, DY 4M CSSA hamster passage 2; CSSA3, DY 4M CSSA hamster passage 3; PSSA, DY PSSA hamster PMCA round 3; PSSA1, DY PSSA hamster passage 1.





**Supplemental Figure 2. PMCA conversion efficiency of hamster prion strains.**

Western blot analysis of intraspecies PMCA reactions seeded with tenfold serial dilutions of A) high conversion efficiency prion strains HY TME, HaCWD and 263K scrapie and B) low conversion efficiency prion strains 139H scrapie and ME7H scrapie. Each prion sample was examined with 3-5 biological replicates consisting of 4 technical replicates each. Uninfected (UN) brain homogenate or HY TME-infected brain homogenate seeded PMCA reactions were included in each experiment as negative and positive controls, respectively.


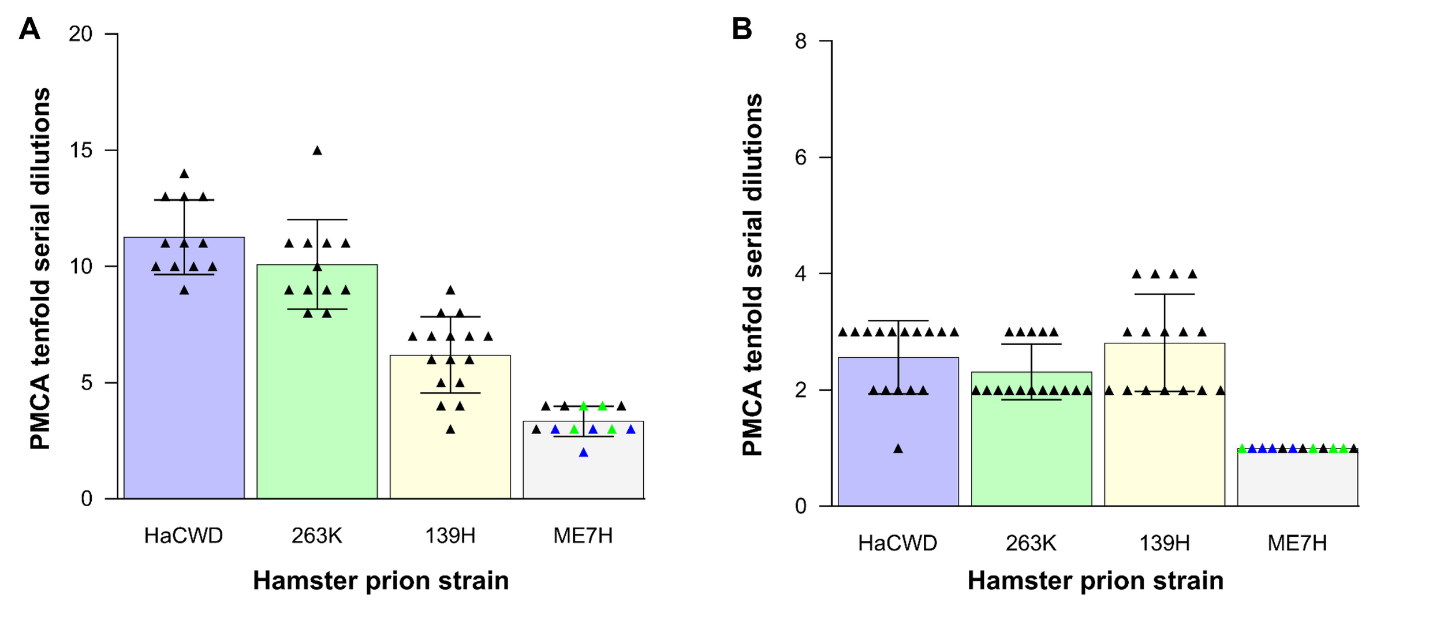


**Supplemental Figure 3.** Quantification of PMCA conversion efficiency of endpoint titration of tenfold serial dilutions of brain homogenate seeded into A) intraspecies or B) interspecies PMCA reactions in mouse brain substrate. Bar graphs represent all technical replicates for each prion sample, with means and standard deviations. *Abbreviations:* HaCWD, hamster-adapted chronic wasting disease; 263K, 139H or ME7H hamster-adapted scrapie.





**Supplemental Figure 4. Antibody PRC7 binds to mouse PrP, but not to hamster PrP.** Western blot analysis of PRC7 antibody affinity for PrP in mice or hamsters with or without PK digestion. *Abbreviations:* UN_Mo_, uninfected mouse; RML, Rocky Mountain laboratory mouse-adapted scrapie; UN_Ha_, uninfected hamster; HY, hyper hamster-adapted TME; DY, drowsy hamster-adapted TME; CSSA1, DY 4M CSSA hamster passage 1; CSSA2, DY 4M CSSA hamster passage 2; CSSA3, DY 4M CSSA hamster passage 3.
